# Supplementary material for: Feasibility of serial measurement of nitrite for pharmacodynamic monitoring and precision prescribing in urinary tract infections
Source: Commun Med (Lond). 2025 Jul 1;5:268. doi: 10.1038/s43856-025-00969-6 (PMC12217021; doi:10.1038/s43856-025-00969-6)
Supplement: Supplementary file 1 — Supplementary Information [file 43856_2025_969_MOESM1_ESM.pdf]

Feasibility of serial measurement of nitrite for pharmacodynamic monitoring and precision prescribing in urinary tract infections – supplementary file

Authors:

Ellen V Stadler[1,2,3], Alison Holmes[1,2,3,4,5], Danny O`Hare[1,6], Mark Sutton[7,8], Colin Brown[9], Timothy M Rawson[1,3,4]

Affiliations:

1: Centre for Antimicrobial Optimisation, Imperial College London, London, UK.

2: Department of Infectious Diseases, Imperial College London, London, UK.

3: National Institute for Health Research, Health Protection Research Unit in Healthcare Associated Infections and Antimicrobial Resistance, Imperial College London, London, UK.

4: David Price Evans Infectious Diseases & Global Health Group, The University of Liverpool, Liverpool, United Kingdom.

5: Fleming Institute, Imperial College London, London, UK

6: Department of Bioengineering, Imperial College London, London, UK.

7: Antimicrobial Discovery, Development and Diagnostics (AD3) UK Health Security Agency, Porton Down, Salisbury, Wiltshire, UK

8: Institute of Pharmaceutical Science, King's College London, London, UK

9: Healthcare Associated Infections, Fungal, Antimicrobial Resistance, Antimicrobial Use, and Sepsis Division, UK Health Security Agency, London, UK

Corresponding author:

Ellen V Stadler; Centre for Antimicrobial Resistance, Imperial College London, Hammersmith Campus, Du Cane Road, London W120NN, UK; Tel: 44-77-0903-72-72; E-mail: ellen.stadler20@imperial.ac.uk

Supplementary Figure 1: **Correlation of generated nitrite and bacterial count in routinely collected clinical urine samples of patients with and without confirmed UTI, not corrected for creatinine**

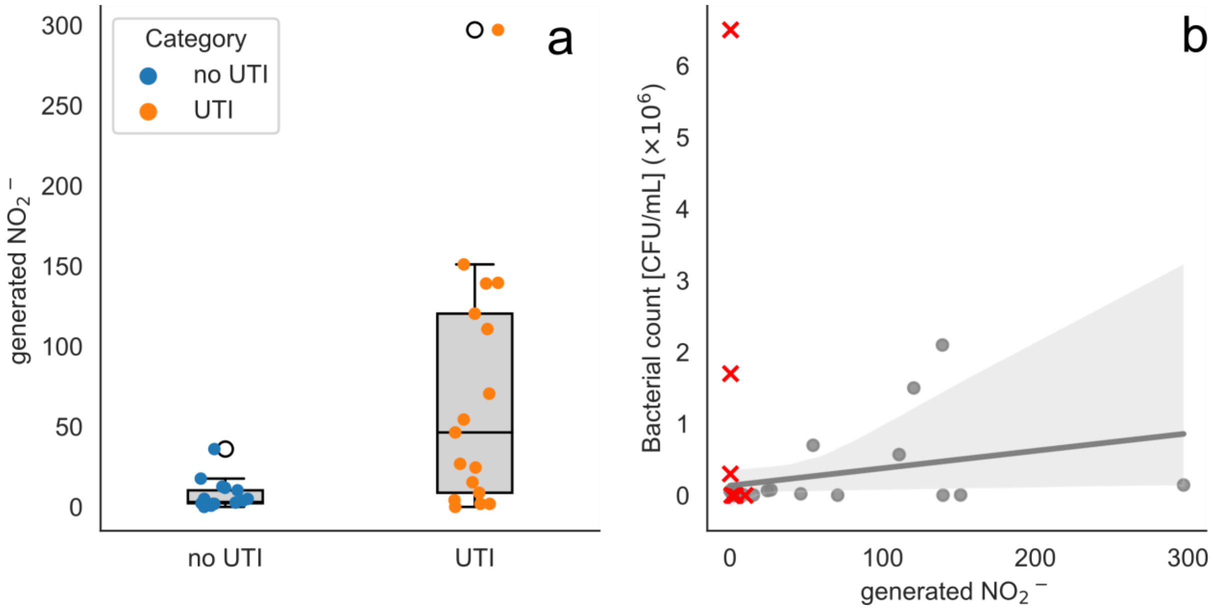

a) Absolute generated nitrite for 25 patients without UTI and 25 patients with confirmed UTI. The black dots represent the mean values for each group, with whiskers showing the 95% confidence intervals. Mann-Whitey U-test test reveals a significant difference between generated nitrite in no UTI vs UTI patients (U = 75.5 and p-value = 0.004). b) The absolute generated nitrite for UTI patients is not significantly correlated with bacterial count, with the solid line showing the linear least squares fit and the shaded area representing the 95% confidence interval. Spearman correlation reveals a spearman coefficient of 0.48 and p-value of 0.051. Red points indicate UTI samples that tested false low for nitrite and were excluded from the analysis (nitrite <20 uM and CFU/mL >0.2x10^6, red crosses).
