# Supplementary material for: Feasibility of serial measurement of nitrite for pharmacodynamic monitoring and precision prescribing in urinary tract infections
Source: Commun Med (Lond). 2025 Jul 1;5:268. doi: 10.1038/s43856-025-00969-6 (PMC12217021; doi:10.1038/s43856-025-00969-6)
Supplement: Supplementary file 2 — Description of Additional Supplementary Files [file 43856_2025_969_MOESM2_ESM.docx]

Description of Additional Supplementary Files

**File name:** Supplementary Data 1

**File description:** The source data of Figure 1 and Figure 2

**File name:** Supplementary Data 2

**File description:** The source data of Figure 3 and Figure 4
