# Supplementary material for: Feasibility of serial measurement of nitrite for pharmacodynamic monitoring and precision prescribing in urinary tract infections
Source: Commun Med (Lond). 2025 Jul 1;5:268. doi: 10.1038/s43856-025-00969-6 (PMC12217021; doi:10.1038/s43856-025-00969-6)
Supplement: Supplementary file 5 — Reporting summary [file 43856_2025_969_MOESM5_ESM.pdf]

nature portfolio

Corresponding author(s): Ellen Stadler

Last updated by author(s): Apr 7, 2025

Reporting Summary

Nature Portfolio wishes to improve the reproducibility of the work that we publish. This form provides structure for consistency and transparency in reporting. For further information on Nature Portfolio policies, see our Editorial Policies and the Editorial Policy Checklist.

Statistics

For all statistical analyses, confirm that the following items are present in the figure legend, table legend, main text, or Methods section.

n/a	Confirmed	
☐	☒	The exact sample size (*n*) for each experimental group/condition, given as a discrete number and unit of measurement
☐	☒	A statement on whether measurements were taken from distinct samples or whether the same sample was measured repeatedly
☐	☒	The statistical test(s) used AND whether they are one- or two-sided<br>*Only common tests should be described solely by name; describe more complex techniques in the Methods section.*
☒	☐	A description of all covariates tested
☐	☒	A description of any assumptions or corrections, such as tests of normality and adjustment for multiple comparisons
☐	☒	A full description of the statistical parameters including central tendency (e.g. means) or other basic estimates (e.g. regression coefficient) AND variation (e.g. standard deviation) or associated estimates of uncertainty (e.g. confidence intervals)
☐	☒	For null hypothesis testing, the test statistic (e.g. *F*, *t*, *r*) with confidence intervals, effect sizes, degrees of freedom and *P* value noted<br>*Give P values as exact values whenever suitable.*
☒	☐	For Bayesian analysis, information on the choice of priors and Markov chain Monte Carlo settings
☒	☐	For hierarchical and complex designs, identification of the appropriate level for tests and full reporting of outcomes
☐	☒	Estimates of effect sizes (e.g. Cohen's *d*, Pearson's *r*), indicating how they were calculated

Our web collection on statistics for biologists contains articles on many of the points above.

Software and code

Policy information about availability of computer code

Data collection	Data collection for colorimetric detection of analytes was performed with BMG Labtech MARS data analysis software v5.02 R3.
Data analysis	Python v3.12. Custom code is available upon reasonable request.

For manuscripts utilizing custom algorithms or software that are central to the research but not yet described in published literature, software must be made available to editors and reviewers. We strongly encourage code deposition in a community repository (e.g. GitHub). See the Nature Portfolio guidelines for submitting code & software for further information.

Data

Policy information about availability of data

All manuscripts must include a data availability statement. This statement should provide the following information, where applicable:

- Accession codes, unique identifiers, or web links for publicly available datasets
- A description of any restrictions on data availability
- For clinical datasets or third party data, please ensure that the statement adheres to our policy

All data supporting the findings of this study are available within the paper. The source data of published figures is publicly available on figshare under the following link: https://figshare.com/projects/
Serial_measurement_of_nitrite_for_pharmacodynamic_monitoring_and_precision_prescribing_in_urinary_tract_infections_A_proof_of_concept_-
_Source_Data/243896

Research involving human participants, their data, or biological material

Policy information about studies with human participants or human data. See also policy information about sex, gender (identity/presentation), and sexual orientation and race, ethnicity and racism.

Reporting on sex and gender	This study included 25 female patients with confirmed E. coli urinary tract infections (UTIs) and 25 female patients without UTIs. The design focused on UTI biomarkers and antimicrobial response in women, as UTIs are more common in this group. No analysis was performed based on sex or gender, as only female participants were included, and gender identity was not collected. Ethical approval and consent for data sharing were obtained, but no disaggregated sex or gender data were collected.
Reporting on race, ethnicity, or other socially relevant groupings	This study did not involve the use of socially constructed or socially relevant categorization variables such as race, ethnicity, or socioeconomic status. The participants were categorized solely based on the presence or absence of a urinary tract infection (UTI) and biological sex (female), which was determined based on medical records. These variables were chosen for their relevance to the clinical focus of the study, which examined nitrite as a biomarker for UTI progression and antimicrobial response. Since there were no variables like race or socioeconomic status involved, confounding variables were not specifically controlled for in the analysis. The focus remained on microbiological and biochemical factors, such as nitrite levels and bacterial counts.
Population characteristics	The study included 50 female participants, divided into two groups: 25 with confirmed urinary tract infections (UTIs) and 25 without UTIs. The age range of participants was not specified in the manuscript. All UTI cases involved uncomplicated infections caused by E. coli, with confirmed bacterial growth (>105 CFU/mL) and white cell count (WCC) >50-100. Participants in the UTI group had no history of prior antibiotic treatment within the 48 hours before sample collection. The non-UTI group had no suspected infections, with no bacterial growth and a WCC <50. Past diagnoses or treatments were not specifically mentioned but were excluded based on the study's inclusion criteria. Genotypic information and treatment categories were not collected for the purpose of this study.
Recruitment	Participants were selected from routine clinical care at Imperial College Healthcare NHS Trust, with urine samples collected from 25 female patients with confirmed urinary tract infections (UTIs) and 25 female patients without UTIs. The selection was based on clear clinical criteria such as bacterial growth and white cell count. This approach ensures that the study is rooted in real-world clinical data, providing valuable insights into UTI biomarkers.While the study sample was limited to females, this choice was intentional and appropriate given the high prevalence of UTIs in this group. Although there could be some self-selection bias—since patients who seek care are likely different from those who do not—the clinical criteria used for sample selection help ensure that the data are relevant and reflective of typical UTI cases. As a result, while these biases are acknowledged, they are unlikely to significantly impact the robustness of the study's findings, which focus on the relationship between biomarkers and UTI progression.
Ethics oversight	The study protocol was approved by the Health Research Authority (HRA) and the Research Ethics Committee (REC), under the COREC application number 06/Q0406/20. The HRA oversees ethical approval and governance of research in the UK, while the REC ensures compliance with ethical standards in studies involving human participants. Link to the ethics protocol: https://www.hra.nhs.uk/planning-and-improving-research/application-summaries/research-summaries/microbial-products-and-their-effects-on-the-patient-version-1-covid-19/

Note that full information on the approval of the study protocol must also be provided in the manuscript.

Field-specific reporting

Please select the one below that is the best fit for your research. If you are not sure, read the appropriate sections before making your selection.

☒ Life sciences ☐ Behavioural & social sciences ☐ Ecological, evolutionary & environmental sciences

For a reference copy of the document with all sections, see nature.com/documents/nr-reporting-summary-flat.pdf

Life sciences study design

All studies must disclose on these points even when the disclosure is negative.

Sample size	The sample size for this in-vitro study was determined based on the exploratory nature of the proof-of-concept research and informed by retrieved studies from the relevant literature on comparable methodologies. Thus, sample size calculation was not carried out.
Data exclusions	3 data points were exluded in analysis in Figure 3), based on criteria defined in the main text of the paper, namely false low nitrite levels defined by nitrite <1uM and CFU/mL >0.2x106^6.
Replication	Experiments in the in-vitro study (Fig 1 and 2) were run in triplicates.
Randomization	Randomization for different experimental groups was not relevant as samples were generated (artificial urine spiked with two different bacterial strains). Allocation to UTI and non-UTI groups for the human samples was performed according to specific criteria (confirmed growth of E. coli >105 CFU/mL, white cell count (WCC) > 50-100, no epithelial cells for UTI and no suspected infection, no bacterial growth, WCC < 50, no epithelial cell for non UTI) and randomization was therefore not applicable.
Blinding	Investigators were not blinded for the in vitro study and human sample analysis.

Reporting for specific materials, systems and methods

We require information from authors about some types of materials, experimental systems and methods used in many studies. Here, indicate whether each material, system or method listed is relevant to your study. If you are not sure if a list item applies to your research, read the appropriate section before selecting a response.

Materials & experimental systems

n/a	Involved in the study	
☒	☐	Antibodies
☒	☐	Eukaryotic cell lines
☒	☐	Palaeontology and archaeology
☒	☐	Animals and other organisms
☒	☐	Clinical data
☒	☐	Dual use research of concern
☒	☐	Plants

Methods

n/a	Involved in the study	
☒	☐	ChIP-seq
☒	☐	Flow cytometry
☒	☐	MRI-based neuroimaging

Plants

Seed stocks	n/a
Novel plant genotypes	n/a
Authentication	n/a
